# Qualitative process evaluation of an Australian alcohol media literacy study: recommendations for designing culturally responsive school-based programs

**DOI:** 10.1186/s12889-017-4031-3

**Published:** 2017-02-02

**Authors:** Chloe S. Gordon, Lisa K. Kervin, Sandra C. Jones, Steven J. Howard

**Affiliations:** 1 Early Start Research Institute, University of Wollongong, Wollongong, 2520 Australia; 2 Centre for Health and Social Research, Australian Catholic University, Melbourne, 3065 Australia

**Keywords:** Process evaluation, Alcohol, Media literacy, Cultural context, School, Australia, Children

## Abstract

**Background:**

Alcohol media literacy programs seek to mitigate the potentially harmful effects of alcohol advertising on children’s drinking intentions and behaviours through equipping them with skills to challenge media messages. In order for such programs to be effective, the teaching and learning experiences must be tailored to their specific cultural context. Media in the Spotlight is an alcohol media literacy program aimed at 9 to 12 year old Australian children. This study evaluates the process and implementation of the program, outlining the factors that facilitated and inhibited implementation. From this evaluation, a pedagogical framework has been developed for health professionals implementing culturally responsive programs in school settings.

**Methods:**

Process measures included: semi-structured interviews with teachers before and after the program was implemented (*n* = 11 interviews), program evaluation questionnaires completed by children (*n* = 166), lesson observations completed by teachers (*n* = 35 observations), and reflective journal entries completed by the researcher (*n* = 44 entries). A thematic analysis approach was used to analyse all of the data sets using NVivo. Inductive coding was used, whereby the findings were derived from the research objectives and multiple readings and interpretations of the data.

**Results:**

Five key pedagogical considerations were identified that facilitated implementation. These were: connecting to the students’ life worlds to achieve cultural significance; empowering students with real-world skills to ensure relevance; ensuring programs are well structured with strong connections to the school curriculum; creating developmentally appropriate activities while providing a range of assessment opportunities; and including hands-on and interactive activities to promote student engagement. Three potential inhibitors to implementing the alcohol media literacy program in upper-elementary school classrooms were identified. These included topic sensitivities, classroom management challenges, and fitting new programs into already busy school schedules.

**Conclusion:**

Overall, the program content and individual lessons were well received by the teachers and students. The lessons learned from the development, implementation and evaluation of this program can provide health professionals with key pedagogical strategies for designing culturally responsive educational programs. Culturally responsive programs are critical for ensuring interventions are effective for their specific context.

## Background

There are significant physical, mental and social consequences that can result from underage drinking, such as impaired memory and brain functioning, accidents, injuries, violence, risky sexual behaviour and self-harm [1–3]. Childhood (6–12 years of age) is a critical period when alcohol expectancies begin to form [4], and children are most cognitively vulnerable to the persuasive appeals of advertising. Research has demonstrated a strong correlation between children’s exposure to alcohol advertising, positive alcohol expectancies, drinker identity and future drinking [5–7]. A presumably simple solution to reducing children’s future drinking would be to reduce their exposure to alcohol advertising. Yet this solution becomes highly improbable given the current alcohol marketing landscape, with limited and ineffective regulation in countries such as Australia, New Zealand, Canada and the UK [8, 9]. Children are exposed, and receptive, to high levels of advertising through avenues such as television [10, 11], movies [12], branded merchandise [7, 13] and, more recently, social media [14].

Media literacy education, which involves students accessing, analysing, evaluating, and creating media in a variety of forms [15, 16], is gaining momentum as an approach to substance abuse prevention in schools [17]. A number of alcohol media literacy (AML) programs have been developed to challenge potentially harmful media messages and subsequently influence health behaviours and attitudes [18–24]. These programs have demonstrated positive outcomes on a range of measures including a decreased preference for alcohol branded merchandise (a precursor to drinking behaviour), increased media deconstruction skills, and lowered drinking intentions [25].

While AML programs have made a valuable contribution to the alcohol prevention field [18–25], the majority have been developed in the US and are therefore relevant to that specific context. There is a need for AML programs to be developed for other contexts, with specific attention to jurisdiction-specific alcohol marketing regulation and cultural nuances [25]. This is particularly important given that youth are more likely to drink the specific alcohol brands to whose advertising they are most exposed [26]. The critical importance of cultural considerations was demonstrated by the alcohol prevention programme Project Northland [27] which was highly successful with predominantly white, lower-middle class to middle-class youth [28], but unsuccessful when implemented with urban, low-income and multi-ethnic youth [29]. Similarly, another study [30] highlighted the unique context of Northern Mexico when implementing a set of drug-resistance strategies that had been initially developed for US adolescents. The study concluded that prevention programs are more likely to be effective when they have been adapted to be relevant to the specific context.

The Australian advertising scene is replete with depictions of Australian cultural stereotypes such as the ‘larrikin’ male sharing a beer with mates at the beach or over a BBQ [31, 32]. For example, a content analysis of alcohol advertisements aired over 2 months in major Australian cities [33] found that approximately half of the advertisements featured the themes of humour (56.7%) or friendship/mateship (41.5%). Portrayals of friendship/mateship often included instances of belittlement, such as a series of advertisements for Victoria Bitter beer where friends ‘saved’ each other by stopping them from engaging in unmanly behaviours including using hand cream and getting a fake tan. Numerous advertisements also linked alcohol to sport such as Australian football. Similarly, female-targeted advertising increasingly portrays drinking as a way for women to bond with their friends and demonstrate their independence from men [34].

Existing AML programs have also focused solely or primarily on print advertisements, rather than teaching students skills in analysing the broad range of multimodal alcohol advertisements to which they are exposed in Australia and elsewhere [25]. Multimodal advertisements utilise more than one mode, such as television advertisements which combine visual, audio, spatial and gestural information to be processed [35].

Given these gaps in the evidence base, Media in the Spotlight, an AML program for Australian children, was developed. While there are cultural differences within the Australian ‘context’, this study sought, as a preliminary step, to tailor the program to the broader Australian context. The program was contextually tailored through inclusion, analysis and critique of Australian alcohol advertisements, Australia media clips, and the values promoted through these advertisements, such as associating alcohol with mateship, sport and larrikinism. Unlike US based AML programs [18–24], the program did not include tobacco advertisements given that advertising for tobacco is completely banned in Australia. Therefore, students’ only exposure to tobacco advertising would be through the program.

Findings from a quasi-experimental trial of the ten-lesson interactive program demonstrated positive results on a range of measures including an increase in media deconstruction skills, decreased perception of social norms for teen drinking and decreased alcohol expectancies [36]. These scales were all derived from existing scales with acceptable levels of reliability and validity. There were a few minor changes to the language used in the scales to ensure contextual relevance. For example, in some instances the word ‘beer’ was replaced with the more general word ‘alcohol’ and a statement assessing social norms was worded ‘Australians drink alcohol’ (see [36] for details on the measures used and quantitative evaluation).

It is critical to understand the factors that can increase uptake of such programs in schools and strengthen their sustainability. Previous evaluation research in this area has focussed primarily on quantifying the effectiveness of programs, with limited attention given to the implementation process [25]. Process evaluations can help to explain why a program was or was not effective in a real world setting [37–40], thereby informing the improvement and sustainability of innovative programs in schools [37]. The purpose of this paper is to examine and reflect upon the implementation process of Media in the Spotlight, and through this provide a pedagogical framework for health professionals seeking to design and implement culturally responsive programs in educational settings.

## Method

### Design

This process evaluation included semi-structured interviews with teachers before and after the program was implemented, student evaluation questionnaires, teacher lesson observations, and researcher reflective journal entries. Multiple process measures were integrated into the intervention design to obtain rich data from stakeholders including teachers, students and the program implementer; and the triangulation of data sources was employed to increase study validity [41].

### Participants

Four primary (elementary) schools from a metropolitan area in New South Wales, Australia were allocated to either the intervention group which received the ten AML lessons (*n* = 83 students), or a wait-list control group (*n* = 82 students). The schools had relatively high indexes of community socio-educational advantage (ICSEA), with values ranging from 1040 to 1045 (1000 is the median) [42].

### Procedure

The study protocol was approved by the University Human Research Ethics Committee (HE14/361), the State Education Research Application Process (SERAP no. 2014112) and the participating schools. The program was implemented by the first author of this paper to increase implementation fidelity and delivered to the Grade 5 and 6 classes over a 10 week term, with one lesson taught each week as part of the English (Language Arts) and Personal Development and Health, Physical Education (PDHPE) school curriculum.

### Measures

#### Teacher interviews

Semi-structured, audio-recorded face-to-face interviews were conducted with six Grade 5 and 6 teachers (*n* = 11 interviews) involved in the study, before and after the program was taught, to explore the teachers’ perceptions of the program. Examples of questions used to guide the interviews are: What are your overall impressions of the program? (follow-up questions were: What do you like about the program? What would you change about the program?) Does the program seem easy to implement? (follow-up questions were: What challenges do you think might arise when implementing the program? What features make the program easy to use, if any?). Five teachers participated in the pre-program interviews and six in the post-program interviews. The interview duration ranged from 10 to 36 min. The themes that arose from the teacher interviews were used to guide the coding for the teacher observations and researcher reflective journal entries. The themes were found to be consistent across the three data sources, thereby providing strong support for the themes.

#### Teacher observations

Non-participant observations were completed by the classroom teachers (*n* = 35 observations) as they observed the researcher delivering each lesson. The 35 observations were collected from five teachers across the ten lessons taught depending on which lessons they were present for. The teachers noted how effectively the program was implemented and any classroom management issues that arose while the program was being taught in a real world setting [39] on an observation grid. Prompts on the observation grid included: Student engagement (classroom management issues, level of interest in discussions and questions asked, student enthusiasm), and teaching content (the extent to which the planned lesson material is covered and deviations from the planned content, lesson timing).

#### Program evaluation questionnaires

The students’ (*n* = 165) impressions of the program were obtained through a program evaluation questionnaire that was created for this study and administered at the end of the last AML lesson. The questionnaire included three open ended questions: (1) What did you like most about Media in the Spotlight? (2) What did you like least about Media in the Spotlight? And (3) What was the most important knowledge/skill you learned from Media in the Spotlight?

#### Researcher reflective journal

The reflective journal provided a personal perspective on how effectively the program was implemented and the challenges faced during implementation (*n* = 44 entries). The journal entries were largely unstructured to allow the researcher to freely record any thoughts and feelings on the lessons taught, however were focussed around pedagogical recommendations and inhibitors to implementation.

### Analyses

A thematic analysis approach was used to analyse all of the data sets using NVivo. The first author transcribed the teacher interviews and then imported the data into NVivo. Inductive coding was used, whereby the findings were derived from the research objectives (pedagogical recommendations and inhibitors to implementation) and multiple readings and interpretations of the data themselves rather than an existing coding framework [43]. The coding framework derived from analysis of the interviews was then applied to the student program evaluation questionnaire, teacher observations and researcher reflective journal, thereby employing a deductive method. Credibility checks involved the research team reviewing the potential themes identified by the first author. The research team discussed the meaning of each theme, the supporting evidence, and ways to refine the themes by combining themes together or creating subthemes. The themes were then refined and further reviewed by the research team.

### Brief program description

The content for Media in the Spotlight was based on the Centre for Media Literacy’s [44] five core media literacy concepts: understanding that all media messages are ‘constructed’ using particular techniques; created using a creative language with its own rules; have embedded values and points of view; constructed for a purpose such as to gain power or to entertain; and different people experience the same message differently [44]. The program was also designed to meet outcomes from both the English (Language Arts) and PDHPE (health) Australian primary (elementary) school curriculum, to provide greater time and flexibility to teach the program.

The content and pedagogy for the program was developed and refined through working collaboratively with academics and professionals in education, alcohol research, and social marketing fields; drawing upon considerations from a systematic review of existing programs [25]; and incorporating feedback from a pilot trial [45]. The lessons (each approximately 1 h in duration) provided opportunities for students to work individually, in groups and as a whole class. Each lesson began with an introductory activity to capture interest, elicit prior knowledge and revise concepts and skills taught in previous lessons. The lesson focus balanced explicit instruction with hands-on (practical) activities that utilised authentic texts from their real-world context. Each lesson concluded with a hands-on activity to apply the knowledge and skills learnt.

The program drew upon multimodal Australian advertising examples to ensure representation of the different facets of the advertising culture. Students analysed alcohol ads in sport; considered how alcohol sponsorship of sport can influence attitudes and behaviour; viewed forms of alcohol branded merchandise (ABM); speculated how ABM influences people to buy alcohol; were taught the range of persuasive techniques used to persuade such as colour, size, slogans, good times and attractive people; and then analysed alcohol print and TV ads, identifying specific techniques and hidden messages, and countering the hidden messages with facts about alcohol.

Assessment opportunities were embedded throughout the program. Students were distributed ‘exit slips’ at the end of each lesson; an A4 piece of paper with a question about a key concept taught (formative assessment). Students assessed their peers’ work through recording two positives (‘stars’) and one suggestion for improvement (‘a wish’) on a proforma (peer assessment). The counterad creation task required the students to produce a work sample that drew upon knowledge and skills taught throughout the 10-lesson program (summative assessment).

## Results

### Participant characteristics

Of the 165 students who participated in the study, 9.7% (*n* = 16) identified as Aboriginal or Torres Strait Islander. The majority (64.8%, *n* = 107) identified as having an Australian cultural background. Other cultural backgrounds identified included European (20.6%, *n* = 34), Asian (8.5%, *n* = 14) and New Zealander (3.0%, *n* = 5). The participation rate across schools ranged from 72.6 to 87.5%, with an overall participation rate of 85.1%. Students ranged in age from 9 to 12 years (*M* = 10.81, *SD* = 0.65) and 52.8% were female. The six participating teachers ranged in teaching experience from 3 to 36 years (*M* = 18.83, *SD* = 12.30), with 1 to 12 years experience of teaching students in Grades 5 and 6 (*M* = 5.67, *SD* = 3.78). The six teachers gave informed written consent to participate in the study and publish the provided data.

The themes from the data are organised under two foci: 1) pedagogical recommendations and 2) inhibitors to implementation.

### Pedagogical recommendations

#### Connect the program to the students’ life worlds to achieve cultural significance

All of the teachers identified that the program was meaningful to the students as it connected to their life worlds. The program connected to seminal aspects of the Australian culture, through the inclusion of current and familiar advertisements across a range of advertising platforms, and references to popular sports, leisure activities, and cultural events.

*The ads, they were something that they’ve seen before, it’s now, not old ads, they seem to be ads that they can connect with.* [Teacher, 30 yrs experience, Female]

*I liked all the videos you were showing early, the cricket where they had to count how many ads they saw. I think they enjoyed that and they were really surprised, by maybe how many, the amount of times they saw it, they thought wow, watched cricket before but hadn’t realised.* [Teacher, 16 yrs experience, Male]

*They were excited to create their own ads.* [Teacher observation, lesson 4]

It is important to emphasise that the program did not increase the students’ exposure to, or identification with, the alcohol advertisements, but rather made them critically aware of the presence of these advertisements and their impact on shaping societal values and beliefs.

#### Empower students with real-world skills to ensure relevance

Related to the above-mentioned theme, all of the teachers noted that the program equipped students with skills to critically analyse the alcohol advertisements that they are surrounded by and was therefore relevant to their everyday lives. Again, the additional data sources confirmed the identified theme. About half of the students identified a media literacy skill as the most important component of the program.

*I think they could now look at an ad and probably with their parents walking through a shopping centre and say look at that, what are they trying to do, and I think they’d be able to rattle off what the advertiser is trying to get you to think, which I was pretty impressed by, the level of understanding that they got throughout the 10 weeks and I think they could probably do that now without prompting.* [Teacher, 18 yrs experience, Female]

[I learnt] *that you always have to look for the hidden message and the techniques used and if they’re true or not.* [Student, School 1, program evaluation questionnaire]

*The content in this lesson was great. It really got the students thinking critically and applying their knowledge.* [Teacher observation, lesson 5]

In the context of this study, the students were equipped with skills that translated to their everyday lives.

#### Ensure programs are well structured with strong connections to the school curriculum

In the pre-interviews, all five teachers noted that the program would be easy for a classroom teacher to implement due to its structured and detailed nature. In the post-interviews, all six teachers expressed confidence that they would be able to implement the program as is, with just a few small adjustments to suit the needs of their classes. These adjustments may include providing additional support for a student with specific learning needs to reduce the complexity of a task and aide understanding. For example, the students may be provided with a partially completed proforma for analyzing alcohol ads, where the persuasive techniques used in an advertisement are listed and the student provides examples of the techniques.

In the pre- and post-program interviews, the teachers also noted that the program aligned well with outcomes from both the English (Language Arts) and PDHPE syllabi. The teacher observations and researcher reflective journal affirmed that the program was structured in a way that made it manageable to teach.

*It was really detailed which is really good and obviously very clear the connections that are being made with the syllabus which I think is really helpful as a teacher because as you probably know time is so precious because there’s not much of it and we struggle to fit everything in.* [Teacher, 10 yrs experience, Female]

*The way that you’ve organised it, as I said before, flows, so going from one activity to the next, each following week, I think they’ll sort of gain the knowledge as they go which will be good rather than sort of jumping around. Whereas I feel we’ve actually been involved in a few other research projects and a few of them tend to do that, they jump around a little bit rather than continually building on the information, like building the field.* [Teacher, 3 yrs experience, Female]

*All the lessons up until this one prepared the students for this activity. All students were able to participate and had the prior learning required to make the counter-ad.* [Teacher observation of lesson 9]

In the current study, strong program alignment with the school curriculum and adequate detail were considered valuable components of a school AML program.

#### Create developmentally appropriate activities and provide a range of assessment opportunities

In the post-program interviews, all six teachers reported that they believed the students had achieved the intended learning outcomes due to the developmental appropriateness of the activities. The researcher’s reflective journal also noted that the majority of students appeared to be grasping the intended learning outcomes for each lesson. In either the pre or post-program interviews, four out of the six teachers commented positively about the opportunities that the assessment tasks afforded to provide feedback on student learning, refine teaching practice and report on whether learning outcomes have been met.

*I think it was pitched definitely at the right level. I think they were able to understand all the concepts and everything pretty well.* [Teacher, 36 yrs experience, Female]

*One of the best features I liked was your exit slips, I thought that was a really good idea, for them to refocus at the end of the lesson, but also for you as an evaluation tool, ‘did they get what I was hoping to achieve?’ I really like that idea. I think I might use that myself.* [Teacher, 10 yrs experience, Female]

*Loved the post-it note strategy! Great assessment for learning* [Teacher observation of lesson 2]

Including assessments throughout the program allowed the implementer to continually assess whether the students were grasping the key concepts and provide further scaffolding where needed.

#### Include hands-on and interactive activities to promote student engagement

In the pre and post-program interviews, all of the teachers noted that the students were involved in the learning experiences due to the hands-on and interactive nature of the program, providing examples from specific lessons. This was reinforced by the teacher observations and the researcher reflective journal. In the post-program interviews, five of the six teachers identified the creation of the counter-ad, where students changed aspects of an advertisement to reflect a truth about alcohol that was not shown in the original ad, as the most engaging component of the program for the students. In the student program evaluation, the majority of the students identified the hands-on activities, in particular the counter-ad creation, as the best part of the program.

*The counter-ad was fun because we could expose the truth* [Student, School 2, program evaluation questionnaire]

*You’ve obviously tried to use a lot of teaching strategies, not just one way of doing things. The visual literacy stuff is really relevant…and anything in terms of designing stuff the kids love.* [Teacher, 10 yrs experience, Female]

*Because there was a lot of visual, there was a lot of discussion, speaking and listening, there was a lot of communicating going on…it covered all learning, some children like tactile, some children like visual, so it covers all those learning needs.* [Teacher, 30 yrs experience, Female]

*The role play was a good way to engage students.* [Teacher observation of lesson 3]

The counter-ad activity was particularly engaging as it involved the students in creating text that challenged the messages presented in alcohol advertisements.

### Inhibitors to implementation

#### Topic sensitivities

In the pre-program interviews, four of the five teachers flagged the sensitive nature of the alcohol topic as an issue to be mindful of, particularly around student disclosures.

*I suppose one challenge might be because it is talking about alcohol, you may get some kids saying some things…my brother likes to go out and drink, that sort of thing.* [Teacher, 10 yrs experience, Female]

*I’ve got one boy who’ll look to be cheeky whenever he can… like the picture of the girls towards the end there I thought was tastefully appropriate, you could get definitely worse pictures than that, but I’m sure he’ll find something funny to say about it.* [Teacher, 16 yrs experience, Male]

The teacher observations and researcher reflective journal did not note any issues arising from the sensitive nature of the alcohol topic as the program was being implemented. Furthermore, alcohol topic sensitivities were not raised by any of the six teachers in the post interviews, suggesting that it was not a significant issue in the current program. The topic was approached in a way that connected to the students’ lifeworlds, using the advertisements that they are surrounded by as a springboard for exploring problematic alcohol usage. While the topic is sensitive, it is mandated in the school curriculum. The schools and teachers were therefore in agreement that the topic needed to be taught to the children. The advertisements included in the program were carefully selected and only those which students would have already been exposed to were included. For example, it was decided to not include alcohol brand pages on Facebook given that the students are below the legal age for Facebook usage.

#### Classroom management issues

In the pre and post-program interviews, none of the teachers foresaw any significant challenges in implementing the program, although they flagged the usual everyday classroom management challenges, and the challenge of the implementer not knowing the children. The teacher observations and researcher reflective journal also noted everyday classroom management challenges.

*No [major challenges]. Probably the challenges I saw were more from the point of view of the kids not being used to your teaching style and you not knowing them as well.* [Teacher, 18 yrs experience, Female]

*There were a few that might have had a bit of difficulty…they struggle to pay more attention…but that’s more them, not so much the content you were teaching, because I know what their behaviour is normally like…* [Teacher, 3 yrs experience, Female]

*The students seemed engaged throughout the whole lesson. They were a little fidgety towards the end, maybe an “out of seats” activity could have helped engagement.* [Teacher observation, lesson 2]

Classroom management issues will always be present to an extent, however they can be minimised through implementing the aforementioned pedagogical considerations. As highlighted by one of the teachers, some classroom management issues may be alleviated by the regular classroom teacher implementing the program as they have a thorough knowledge of individual student needs and capabilities.

#### Time constraints

In the post-program interviews, two of the six teachers identified lack of time as a potential barrier to implementation due to the competing demands present in elementary school.

*Time. Just time, like everything else. It’s one of those things that things happen, days happen…that just take away from your time and just to get through every single thing, may or may not be an issue depending upon what else is going on in the school.* [Teacher, 18 yrs experience, Female]

*I think being in fourth term, there’s so much going on. In a way in Year 5 it is good because they’re a bit more mature, Year 6 maybe earlier, maybe third term heading towards high school.* [Teacher, 30 yrs experience, Female]

For the current program, each lesson addressed outcomes from two curriculum areas. This integration of subject matter also afforded more time and flexibility for teaching the program.

## Discussion

This study evaluated the process and implementation of Media in the Spotlight, an interactive AML program created for a specific cultural context. The study aimed to provide a pedagogical framework for health professionals implementing culturally responsive programs in school settings. In a time of significant curriculum change and competing demands for elementary school teachers [46], it is valuable to understand what teachers consider to be important in a health program, to increase program uptake and sustainability in schools. In drug and alcohol education especially, it is critical for programs to be created in a way that is culturally responsive, to ensure program relevance and effectiveness [25].

Overall, the program was well received by the teachers and feedback suggested that they were open to a cross disciplinary approach to alcohol prevention in schools. The teachers did not report any significant inhibitors to implementation apart from classroom management issues and the time constraints imposed by an overcrowded curriculum. The issue of time constraints in an “overcrowded curriculum” is commonly cited as a problem for teachers [47, 48]. However, teacher uptake and sustainability of programs can be increased through developing a structured program that is easy to follow, has clear and strong connections to the school curriculum, embeds assessment opportunities within the lessons and includes achievable outcomes.

The teachers also expressed confidence in being able to implement the program themselves due to the program’s structured and comprehensive nature. A well-structured program that is achievable, interactive and relevant for students will also keep students engaged and reduce the likelihood of classroom management issues [49, 50]. Given the demanding nature of the teaching profession, teachers value having all necessary resources included within programs so that they do not have to spend time sourcing information.

The qualitative data sources indicated that the program was enjoyable and acceptable to the students, with the hands-on activities identified as a key strength of the program. This finding is supported by education learning theories such as constructivism which posits that students learn through constructing knowledge and meaning from their experiences [51]. Further, hands-on and interactive activities that engage students in the topic, and enable them to demonstrate and apply their knowledge, are more likely to sustain student interest [51]. The inclusion of hands-on activities are particularly important for drug and alcohol education, as the most effective programs go beyond transmitting knowledge to teaching skills and building coping strategies [52]. Future programs could consider including related extension activities for students that grasp the concepts quickly. This may be more feasible to achieve when the regular classroom teacher is implementing the program, as they would have an understanding of the needs and capabilities of individual students in the class.

Using culturally bound advertisements as an entry point to learning about alcohol proved powerful, as it connected to the students’ life worlds [53] through use of authentic text from contexts they were familiar with. The media plays a significant role in young people’s lives and can therefore be an effective way of creating relevance and motivation in lessons [54]. The media literacy skills acquired can also enable students to resist the emotional messages presented through ads and positively influence behaviour change [21, 55, 56]. Meta-analyses of school-based drug prevention programs found that skills rather than knowledge are powerful for changing behaviour [57]. This principle, of drawing upon students’ lifeworlds and empowering them with practical skills, can be applied to other sensitive health areas such as sexual health [58] and body image [59]. Of critical importance is ensuring that the stimuli selected is developmentally appropriate and specific to the children’s cultural context.

There are several limitations that should be considered when interpreting the results of this study. The teacher process evaluation data was collected from six teachers in four schools and therefore may not be representative of the wider teaching population, although opinions do represent a range of teaching experiences from early career to highly experienced teachers. Additionally, there may have been a bias against negative disclosure as the interviewer was also the intervention creator and deliverer. However this may have also helped disclosure as the interviewer had developed a rapport with the teachers. Cultural considerations may need to be more narrowly defined to specific regions within a country. Media in the Spotlight was developed, implemented and evaluated in an area with a high percentage of white Anglo-Saxon Australians. In other areas of Australia with more diverse ethnicities, the cultural considerations may differ. Nonetheless, the principles from the pedagogical framework can be applied to other contexts.

## Conclusions

There are significant benefits in early prevention [60], where issues such as underage drinking are prevented before the problem occurs. In order for these programs to be effective, they must be tailored to the cultural context in which they are being implemented. This paper describes the development and implementation of a school-based AML program for Australian upper-elementary school students, and provides a pedagogical framework for health educators when developing programs for different cultural contexts. Key principles include connecting to the students’ life worlds to achieve cultural significance; empowering students with real-world skills to ensure relevance; ensuring programs are well structured with strong connections to the school curriculum; creating developmentally appropriate activities while providing a range of assessment opportunities; and including hands-on and interactive activities to promote student engagement. Potential inhibitors to implementation include topic sensitivities, classroom management challenges, and fitting programs into busy school schedules. However these inhibitors can be minimised through applying the above pedagogical considerations.

## Abbreviations

ABM: Alcohol branded merchandise
AML: Alcohol media literacy
PDHPE: Personal Development and Health, Physical Education
